# Evolving Dynamic S-Boxes Using Fractional-Order Hopfield Neural Network Based Scheme

**DOI:** 10.3390/e22070717

**Published:** 2020-06-28

**Authors:** Musheer Ahmad, Eesa Al-Solami

**Affiliations:** 1 Department of Computer Engineering, Jamia Millia Islamia, New Delhi 110025, India; 2 Department of Information Security, University of Jeddah, Jeddah 21493, Saudi Arabia; eaalsulami@uj.edu.sa

**Keywords:** dynamic S-box, block cryptosystem, fractional Hopfield neural network, security

## Abstract

Static substitution-boxes in fixed structured block ciphers may make the system vulnerable to cryptanalysis. However, key-dependent dynamic substitution-boxes (S-boxes) assume to improve the security and robustness of the whole cryptosystem. This paper proposes to present the construction of key-dependent dynamic S-boxes having high nonlinearity. The proposed scheme involves the evolution of initially generated S-box for improved nonlinearity based on the fractional-order time-delayed Hopfield neural network. The cryptographic performance of the evolved S-box is assessed by using standard security parameters, including nonlinearity, strict avalanche criterion, bits independence criterion, differential uniformity, linear approximation probability, etc. The proposed scheme is able to evolve an S-box having mean nonlinearity of 111.25, strict avalanche criteria value of 0.5007, and differential uniformity of 10. The performance assessments demonstrate that the proposed scheme and S-box have excellent features, and are thus capable of offering high nonlinearity in the cryptosystem. The comparison analysis further confirms the improved security features of anticipated scheme and S-box, as compared to many existing chaos-based and other S-boxes.

## 1. Introduction

With the recent advancements in the field of wired/wireless network communication and electronic sharing of confidential data, the need for information security within the organization has undergone major changes. There is heavy reliance on electronic transfer of confidential data in daily life. Examples include different wallets, ecommerce websites purchases use our debit card info, internet banking and emails, etc. Network security is required to protect data while in transit. Cryptography provides the secure exchange of information between two communicating parties [1]. In cryptography, the plain message is converted to cipher text prior to its being transferred over the communication network, so that the observers or the attackers cannot interpret the original information. Thus, in order to successfully encrypt the message, we need to form a secure cipher from the plain text. To form a secure cipher confusion and diffusion are two essential properties needed, as identified by Claude Shannon. In confusion, each bit of the cipher text depends upon several parts of the key hiding the relationship between the two, therefore making it hard to find the key even if someone has a long plain text. In diffusion, the output bits depend upon the input bits in a complex way: If we change a single bit in the plain text, then at least half of the bits in the output cipher text should vary, and, similarly, if we change one of the bits in the cipher text, then more than half of the bits in the input plain text should change. Therefore, this makes the relationship between the plain text and the cipher text complex [2,3,4].

Modern cryptographic applications extensively make use of block encryption algorithms. Strong block encryption algorithms can be designed with the help of substitution-boxes (S-boxes). In cryptographic systems, the encryption process relies on the nonlinear mapping of plain data to the secure encrypted data. The nonlinear transformation of data is facilitated by the application of the substitution process embedded in the cipher [5,6]. S-box is the only nonlinear component for many block encryption algorithms like IDEA (International Data Encryption Algorithm), DES (Data Encryption Standard), and AES (Advanced Encryption Standard). S-boxes are responsible for creating confusion in data during the encryption process; due to this property of the S-box, it is the essential component of numerous block ciphers. For this reason, the cryptanalyst generally targets the S-box component of the block encryption algorithm; hence, the S-box construction methods have received significant importance and are an active area of research [7,8,9,10].

Basically, an 8 × 8 sized substitution-box indicates that the S-box is having 8 input bits, and 8 output bits can be viewed as a transformation *S*: {0, 1}^8^ → {0, 1}^8^, i.e., it is a one-to-one mapping wherein each 8-bit input is uniquely mapped to a distinct 8-bit output. Therefore, the S-boxes can also be viewed as multi-input and multi-output Boolean functions. Thus, an 8 × 8 S-box inherently comprises of eight candidate Boolean functions. Each function takes 8-bit input and yields 1-bit as output; thereby, all eight Boolean functions collectively give output bits-streams of size 8-bit. The block ciphers make use of two types of S-boxes, namely the static and dynamic S-boxes [11]. The former remains unchanged during every operation of a block cipher, as it is independent to the cipher’s secret key. However, the dynamic S-boxes may be changed due to change in one or more secret key components. Thus, the dynamic S-boxes are highly key-dependent and make the ciphers stronger and more robust against different cryptanalysis, compared to the ciphers, which are based on static S-boxes. As a result, the secure dynamic S-boxes have a dominant part to decide the forte of block cryptosystems. The security features of S-boxes are of vast significance for the security of cryptosystems [12,13,14]. Henceforth, the progress of dynamic, potent, and robust S-boxes is of supreme impact for researchers aspiring to develop strong modern cryptosystems.

Dynamical systems have various applications in numerous disciplines, including cryptology, meteorology, physics, and engineering. They deal with analyses, design, and performance assessment of continuous or discrete nonlinear systems that are extremely subtle to initial conditions. The deterministic nonlinear systems which possess good sensitivity to initial conditions are said to exhibit chaotic behavior [15,16]. If this initial information is not available, the system appears pseudo-random to the observer. The designing of dynamical chaotic systems-based schemes for network security has been a growing area of research in recent years. In chaos-based cryptography, the objectives of a cryptographic system are to obscure information present in the plain text in order to secure the encrypted data. The integral part of creating the confusion is the inception of pseudo-randomness in data at the output. Many block ciphers have been proposed during the last few decades; S-boxes are one of the essential components of block cryptosystems. There are many other approaches, as well, to bring about uncertainty, but dynamical systems have developed to be one of the lucrative choices for creating confusion, generating mix-up, and bringing about nonlinear changes in the data. In recent times, various S-box construction methods have been proposed based on different forms of dynamical models like discrete, continuous, fractional-order, time-delayed, etc. Discrete systems have been used in most of the studies, to avoid the numerical deterioration that may occur in the case of continuous systems. Previously, quantization-based algorithms were used for the construction of S-boxes, using the integer-order continuous-time dynamical systems. More complex systems were used to increase the performance of the S-box component.

Over the past decade, the dynamical behavior of continuous chaotic systems, multi-scroll attractors, and hyperchaotic systems has been intensively studied for engineering and other applications [17]. It has been found out that integer-order nonlinear systems do not show sensitive dependence to initial conditions if the dimension of system is smaller than three [18]. However, in case of fractional-order nonlinear systems, a complicated dynamical behavior is observed even for the system whose dimension is smaller than three. The set of fractional-order dynamical systems is the superset of integer-order systems. Notably, more complicated dynamics and high sensitivity to initial conditions is observed in fractional-order dynamical systems compared to integer-order systems [19]. Dynamics of fractional-order systems are found to be more complex because of their high nonlinearity and non-local character. As a result, the fractional-order systems have been employed in secure communication due to this soothing property. In addition, the incorporation of a time delay in fractional-order systems can further increase the complexity, unpredictability, pseudo-randomness, etc., and can contribute to make strong security primitives [20,21]. Time-delayed systems are applied in a large variety of cases, in many scientific fields, such as feedback loops, artificial neural network, control systems, and application of communications [22]. 

There exist many proposals for S-box construction where the dynamics of integer-order continuous systems (having dimension three or more) are applied. In Reference [23], it was shown that a good S-box can be obtained by using continuous chaotic 3D Lorenz system. In Reference [24], Khan et al. made use of two 3D continuous chaotic systems, namely the Lorenz and Rössler systems, to generate the candidate values of an 8 × 8 substitution-box. Meanwhile, Khan et al. gave a procedure wherein a 3D fractional Rössler chaotic system was applied to synthesis 8 × 8 S-boxes [25]. In Reference [26], the authors constructed an S-box, using a method based on the 3D four-wing autonomous chaotic system. In Reference [27], a continuous six-dimensional hyperchaotic system was explored to generate the initial S-boxes for an Artficial Bee Colony (ABC)-based optimization process which resulted in an optimized S-box. A new S-box design was proposed in Reference [28] which is honorable. The authors’ design was aided with rich dynamic features of a new scaled Zhongtang chaotic system. Islam et al. gave an S-box generation method which was based on a 4D hyperchaotic system in which two pseudo-random 8-bit sequences were obtained to yield an S-box on two-position swapping [29]. Solami et al. [30] designed a random heuristic search method to generate efficient bijective S-boxes by using 5D hyperchaotic system. Wang et al. [31] used a new three-dimensional continuous chaotic map with infinite equilibrium points to design an S-box, but its nonlinearity was also not good enough. Liu et al. [32] proposed employing spatiotemporal chaos to generate S-boxes. They used the non-adjacent coupled map lattices and Arnold’s cat map to extract the spatiotemporal chaotic behavior of the system. However, there exists only one proposal by Özkaynak where the time-delayed models of 1D chaotic Ikeda, sine map, and Logistic are investigated to frame a simple scheme of yielding S-boxes [33], showing satisfactory performances. Similarly, there are only two research works which investigate the fractional-order systems for an S-box design. The first one was given by Özkaynak in Reference [34], which investigated a simple approach of constructing S-boxes by using the 3D fractional Chen system. The second one was suggested by Khan et al. [35], who explored the features of a fractional-order 3D chaotic Rössler system to construct an 8 × 8 S-box. To date, no S-box method has been investigated based on the two-state continuous dynamical system which is of fractional-order and time-delayed as well. The two can also be marked as components of secret key to complicate the work of possible cryptanalysis. The orders of fractional derivatives and amount of time delay extend the possible key space of the security system. In this paper, a new S-box evolution scheme is proposed, using the dynamics of the fractional-order time-delayed two-state Hopfield neural network system. It has been shown that time delay plays a vital role in increasing the nonlinearity of the S-box.

The remaining portion of the paper is maintained as follows. The time-delay fractional Hopfield neural network is discussed in Section 2. Section 3 provides the proposed scheme of initial S-box generation and its evolution for high nonlinearity, using the selected Hopfield system. Section 4 is developed to report the security performance of the proposed S-box under some standard properties, followed by its analyses and comparison with contemporary S-box proposals on dynamical systems. Lastly, Section 5 concludes the research findings of this paper. 

## 2. Fractional-Order Hopfield Neural Network

The two-state time-delayed fractional Hopfield neural network is defined as follows [19].

*D^p^x*(*t*) = *A*_1_*x*(*t*) + *B*_1_*f*(*x*(*t*)) + *C*_1_*f*(*y*(t)) + *D*_1_*f*(*x*(*t* − *τ*)) + *E*_1_*f*(*y*(*t* − *τ*)) + *I*_1_

*D^q^y*(*t*) *= A*_2_*y*(*t*) + *B*_2_*f*(*x*(*t*)) + *C*_2_*f*(*y*(*t*)) + *D*_2_*f*(*x*(*t* − *τ*)) + *E*_2_*f*(*y*(*t* − *τ*)) + *I*_2_

where *x*(*t*) and *y*(*t*) represent the states of the two neurons at time instance, *t*; 0 < *p* and *q* < 1 are the derivative orders; *τ* > 0 denotes the delay introduced in time, *t*; *A*, *B*, *C*, *D*, *E*, and *I* are the associated coefficients corresponding to each state; and *f*(*x*) is the piece-wise linear (PWL) function given as *f*(*x*) = 0.5 × (|*x* + 1| − |*x* − 1|). We followed the procedure as per Grunwald–Letnikov (GL) definition, mentioned in Sections 2.3 and 2.9 in Reference [36], to solve the fractional-order derivative equations of the Hopfield system. The numerical solution of the above two-state time-delayed fractional Hopfield neural network is as follows:

$$\begin{array}{c} x(t_{k})=h^{p}(A_{1}x(t_{k-1})+B_{1}f(x(t_{k-1}))+C_{1}f(y(t_{k-1}))+D_{1}f(x(t_{k}-\tau )+E_{1}f(y(t_{k}-\tau ))+I_{1})-\sum_{j=0}^{k}c_{j}^{\left(p\right)}x(t_{k-j})\\ y(t_{k})=h^{q}(A_{2}y(t_{k-1})+B_{2}f(x(t_{k-1}))+C_{2}f(y(t_{k-1}))+D_{2}f(x(t_{k}-\tau )+E_{2}f(y(t_{k}-\tau ))+I_{2})-\sum_{j=0}^{k}c_{j}^{\left(q\right)}y(t_{k-j}) \end{array}$$

where *h* is the time step, and

$$\begin{array}{ccc} c_{j}^{\left(p\right)}=\left(1-\frac{1+p}{j}\right)c_{j-1}^{\left(p\right)} & & c_{0}^{\left(p\right)}=1; \end{array}$$

$$\begin{array}{ccc} c_{j}^{\left(q\right)}=\left(1-\frac{1+q}{j}\right)c_{j-1}^{\left(q\right)} & & c_{0}^{\left(q\right)}=1; \end{array}$$

Now the transmission delay in time terms are computed though the linear interpolation scheme, expression are as follows (assuming *τ* > *h*).

$$x(t_{k}-\tau )=x(t_{k-d})+\left(\frac{t_{k}-\tau -t_{k-d}}{t_{k-d+1}-t_{k-d}}\right)\left(x(t_{k-d+1})\right)-x(t_{k-d})$$

$$y(t_{k}-\tau )=y(t_{k-d})+\left(\frac{t_{k}-\tau -t_{k-d}}{t_{k-d+1}-t_{k-d}}\right)\left(y(t_{k-d+1})\right)-y(t_{k-d})$$

where *d* = ceil(*τ*/*h*) when *d* ≤ *j* ≤ *n* + 1, and *x*(*t*_k_ − *τ*) = *x*_0_ for 0 ≤ *j* < *d*. After solving the system for pre-specified number of iterations, we get two sequences.

## 3. Proposed S-Box Construction Scheme

The two forms of the time delayed fractional Hopfield neural network with different derivative orders and time delays are identified as System (1) and System (2), given below. The coefficients for the two forms are set at *A*_1_ = −0.25, *A*_2_ = −0.2, *B*_1_ = −0.05, *B*_2_ = 0.02, *C*_1_ = 0.01, *C*_2_ = −0.01, *D*_1_ = −0.01, *D*_2_ = 0.02, *E*_1_ = 0.02, *E*_2_ = 0.01, *I*_1_ = −0.1, and *I*_2_ = 0.4.

**System (1):**

(1) $$\begin{array}{c} D^{p1}x_{1}(t)=-0.25x_{1}(t)-0.05f(x_{1}(t))+0.01f(x_{2}(t))-0.01f(x_{1}(t-\tau_{1}))+0.02f(x_{2}(t-\tau_{1}))-0.1\\ D^{q1}x_{2}(t)=-0.2x_{2}(t)+0.02f(x_{1}(t))-0.01f(x_{2}(t))+0.02f(x_{1}(t-\tau_{1}))+0.01f(x_{2}(t-\tau_{1}))+0.4 \end{array}$$

**System (2)**

(2) $$\begin{array}{c} D^{p2}y_{1}(t)=-0.25y_{1}(t)-0.05f(y_{1}(t))+0.01f(y_{2}(t))-0.01f(y_{1}(t-\tau_{2}))+0.02f(y_{2}(t-\tau_{2}))-0.1\\ D^{q2}y_{2}(t)=-0.2y_{2}(t)+0.02f(y_{1}(t))-0.01f(y_{2}(t))+0.02f(y_{1}(t-\tau_{2}))+0.01f(y_{2}(t-\tau_{2}))+0.4 \end{array}$$

where *x*_1_(0) ≠ *y*_1_(0), and/or *x*_2_(0) ≠ *y*_2_(0), and/or *p*_1_ ≠ *p*_2_, and/or *q*_1_ ≠ *q*_2_, and/or *τ_1_* ≠ *τ*_2_. The two systems are considered as to extend the possible key space of the proposed method. System (1) is utilized to generate the initial configuration of the S-box. However, System (2) is applied to improvise the nonlinearity strength of the initial S-box. The proposed scheme for the highly nonlinear S-box construction is as follows. 

**Step I.** Initialization of S-Box

1. Take initial values of System (1) as *x*_1_(0), *x*_2_(0), *p_1_*, *q_1_*, *τ_1_*, and empty array *S*;
2. Iterate System (1) to obtain *x*_1_ and *x*_2_;
3. Extract w1, w2 from current *x*_1_ and *x*_2_ as
  *w_j_* = [*floor*(*x_j_* × 10^15^)]*mod*(256)  for *j* = 1, 2;
4. Save first occurrence of *w*_1_ and then *w*_2_ in array *S*;
5. Repeat from Step 2 until all distinct 256 elements are recorded in array *S*.

**Step II.** Evolving S-Box Scheme

1. Take initial values of System (2) as *y_1_*(0), *y_2_*(0), *p_2_*, *q_2_*, *τ_2_*, *N*, *itr_max*, *step_x*, *step_y*, *prime_1_*, *prime_2,_* and Δ*τ*;
2. Iterate System (2) for *itr_max* times, to get two arrays, *Y_1_* and *Y_2_*;
3. Extract *U_1_*(*i*), *U_2_*(*i*) from current *Y_1_*(*i*) and *Y_2_*(*i*) as
  *U_j_*(*i*) = [*floor*(*Y_j_*(*i*) × 10^5^)]*mod*(256)  *for j* = 1, 2, and *i* = 1, 2, …, *itr_max*;
4. Append arrays *U_1_* and *U_2_* to get a single array as
  *Q*[1 to *itr_max*] = *U_1_*; *Q*[*itr_max* + 1 to 2 × *itr_max*] = *U_2_*;
5. Compute *rating_1_* = *nonlinearity*(*S*);
6. ***For**k* = 1 to 2 × *itr_max*
    *step_x* = [*step_x* + (*k* × *prime_1_*)*mod*(32)]*mod*(16)
    *step_y* = [*step_y* + (*k* × *prime_2_*)*mod*(32)]*mod*(16)
    *spos* = 16 × *step_x* + *step_y*
    *val* = *S*(*spos*) XOR *Q*(*k*)
    find index *m* such that *S*(*m*) = = *val*
    set *S_1_* = *S*
    *Swap*(*S_1_*(*m*), *S_1_*(*spos*))
    *rating_2_* = *nonlinearity*(*S_1_*)
    ***if*** (*rating_2_* >= *rating_1_*)
       *S* = *S_1_*
       *rating_1_* = *rating_2_*
    ***endif***
  ***endfor***;
7. Increment delay as: *τ*_2_ = *τ*_2_ + Δ*τ*;
8. Repeat Step 2 to Step 7 for *N* number of increments in *τ*_2_.

**Figure 1:**
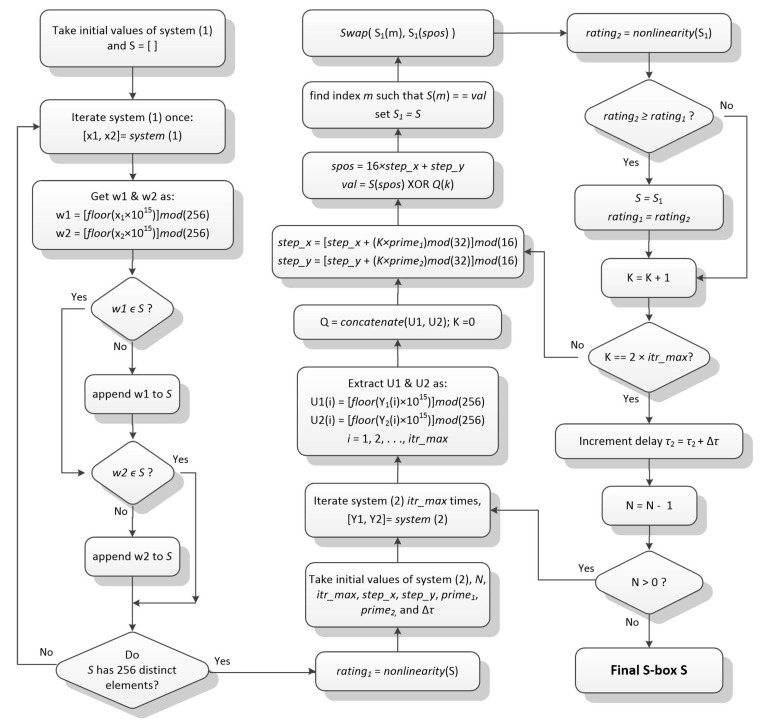
Flowchart of proposed substitution-box (S-box) construction scheme.

The proposed scheme is also illustrated through the flowchart shown in Figure 1.

## 4. Performance Results and Analyses

This section deals with the performance assessment, analyses, and the proposed S-box’s comparison with some state-of-the-art S-box schemes which are based on dynamical systems. Without loss of generality, the settings for simulation of proposed scheme are provided in Table 1. The S-box obtained with these settings, using the proposed scheme, is listed in Table 2. The secret key ***K*** of proposed security scheme includes the components such as ***K*** = (*x*_1_(0), *x*_2_(0), *p*_1_, *q*_1_, *τ_1_*, *y*_1_(0), *y*_2_(0), *p*_2_, *q*_2_, *τ*_2_, *N*, *itr_max*, *step_x*, *step_y*, *prime*_1_, *prime*_2_, and Δ*τ*). All floating-point computations are performed as per the IEEE 754 standard. Thus, the possible key space is more than 2^500^. This enormously large key space is sufficient enough to withstand the brute-force cryptanalysis. The most popular and standard cryptographic properties of S-boxes are as follows: high nonlinearity, low differential uniformity, the strict avalanche criterion equals to 0.5, the satisfaction of bits independence criterion for high bits independence criterion (BIC) nonlinearity and BIC–strict avalanche criterion (SAC) close to 0.5, and low linear approximation probability. A majority of the existing S-boxes schemes have scrutinized their constructed S-boxes mainly against these security properties [37,38,39,40,41]. The following subsections analyzed the proposed S-boxes under the mentioned properties.

### 4.1. Nonlinearity

The nonlinearity measure of a Boolean function, *f*, is computed by knowing the least distance of *f* to the set of all affine functions [42]. Thus, the component Boolean functions of the S-box should have standing nonlinearities scores. The nonlinearity NL(*f*) of any Boolean function f is computed as follows:

$$NL(f)=128\left(1-2^{-8}\underset{z\in {\left\{0,1\right\}}^{8}}{\max}\left|S_{f}(z)\right|\right)$$

$$S_{f}(z)=\sum_{x\in {\left\{0,1\right\}}^{8}}{\left(-1\right)}^{f(x)⊕x.z}$$

where *S_f_*(*z*) is the Walsh–Hadamard transform of Boolean function, *f*. A Boolean function is deemed frail if it tends to have poor nonlinearity. The higher nonlinearity of balanced Boolean functions is considered one of the prominent measures responsible for providing better robustness to any type of linear attack [43]. We find that the nonlinearity scores of the proposed S-box are 110, 110, 112, 112, 112, 110, 112, and 112, which include the minimum *NL* of 110 and an average score of 111.25. Thus, it is very evident that the proposed S-box possesses high nonlinearity performance. The reason being, the proposed scheme made to evolve the S-box based on the nonlinearity rating.

### 4.2. Strict Avalanche Criterion

The strict avalanche criterion was described by Tavares and Webster, and it gets its base on the completeness effect’s notion and the avalanche [44,45]. This criterion measures that by making a single change in input bits, i.e., how many output bits get altered. The SAC is assumed to be satisfied when all the output bits are changed with a likelihood of 0.5, when only one input bit is flipped. Following the procedure given by Webster and Tavares, we get the SAC matrix shown in Table 3. The average of this matrix, which is 0.5007, indicates the SAC value. We can see that this score is close to ideal value of 0.5 with infinitesimal offset of only 0.0007. Thus, the proposed S-box has good avalanche when any of the single input bit is altered and decently satisfies the SAC criterion.

### 4.3. Bits Independence Criterion

The input bits which remain unchanged are explored under the bits independence criterion. The revamping of the independent performance of pair-wise variables of avalanche vectors and unaltered input bits is the asset of this measure. Under this criterion, the avalanche component Boolean functions pairs should be independent to each. It is an effective criterion in symmetric cryptosystem, because by augmenting independence between bits, the recognition and prediction of patterns of the system is not possible [30,46]. Accordingly, the Boolean functions *f* = *f_i_* ⊕ *f_j_* (*i* ≠ *j*) should behave well for nonlinearity and SAC properties both. The BIC performance of the proposed S-box for nonlinearity is shown in Table 4 and for SAC is provided in Table 5. The BIC-nonlinearity (NL) score is 102.57, and BIC–SAC is 0.5034, which indicates the acceptable performance of our S-box under BIC property.

### 4.4. Differential Uniformity

The differential uniformity measures the resistivity of an S-Box against the differential cryptanalysis. The attack procedure of cryptanalysis was given by Biham and Shamir; it is related with developing imbalance on the input/output dissemination to assault block ciphers and S-boxes [47]. Confrontation to this cryptanalysis can be consummate if the Exclusive-OR of each output has identical uniformity with the EX-OR value of each input. If an S-box is uniform in input/output distribution, then it is said to be resistant. It is preferred that the largest value of differential uniformity (DU) in EX-OR table should be as small as possible [48]. The differential uniformity is measured as follows:

$$\delta_{S}=\underset{\Delta x\neq 0,\Delta y}{\max}\left(\#\left\{x\in X|S(x)⊕S(x⊕\Delta x)=\Delta y\right\}\right)$$

where set *X* holds all probable input values, and the cardinality of its elements is 256 for 8x8 S-box. The largest value of EX-OR (differential distribution) table for an S-box should be as small enough to resist the differential cryptanalysis. The differential distribution matrix for the proposed S-box is obtained and is available as Table 6. The highest value of this matrix, i.e., 10, is the differential uniformity for our S-box, and such maximum values are only 7 out of the 256 values in the matrix, thus indicating the good differential uniformity and robustness of the proposed S-box.

### 4.5. Linear Approximation Probability

The method of linear approximation probability (LAP) is helpful in calculating the imbalance of an incident. The largest value of imbalance of an event is measured with the help of the analysis introduced by Matsui [49]. There must be no difference between output and input bits uniformity. Each of the input bits with its results in output bits is examined individually. If all the input elements are 256 for the 8 × 8 S-box, the class of all possible inputs is *d*, and the masks applied on the equality of output and input bits are respectively *m*_x_ and *m*_y_, then maximum linear approximation is the maximum number of the same results and calculated as follows:

$$LAP(S)=\underset{m_{x},m_{y}\neq 0}{\max}\left|\frac{\#\left\{x\in X|x.m_{x}=y.m_{y}\right\}}{256}-0.5\right|$$

A lower value of this measure indicates that S-box is more capable to resist the linear cryptanalysis. The LAP score of proposed S-box is 0.14025.

### 4.6. Comparison

A performance comparison analysis is significant in finding the actual standing of the proposed S-box. Almost all S-box methods were dynamical systems of the following types: (1) high dimensional continuous integer-order systems, (2) time delay system, (3) fractional-order 3D systems, and some other recent S-boxes are opted for the comparison. The performance scores for different security properties of all selected S-boxes are displayed in Table 7.

**Figure 2:**
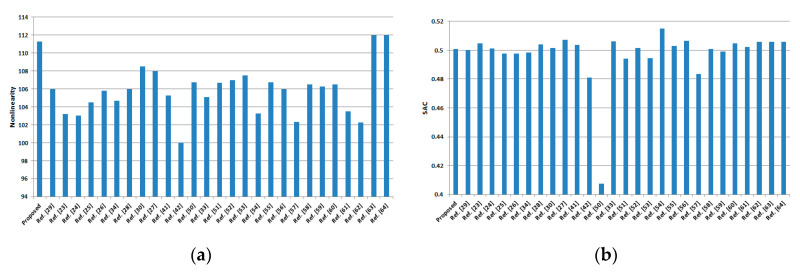

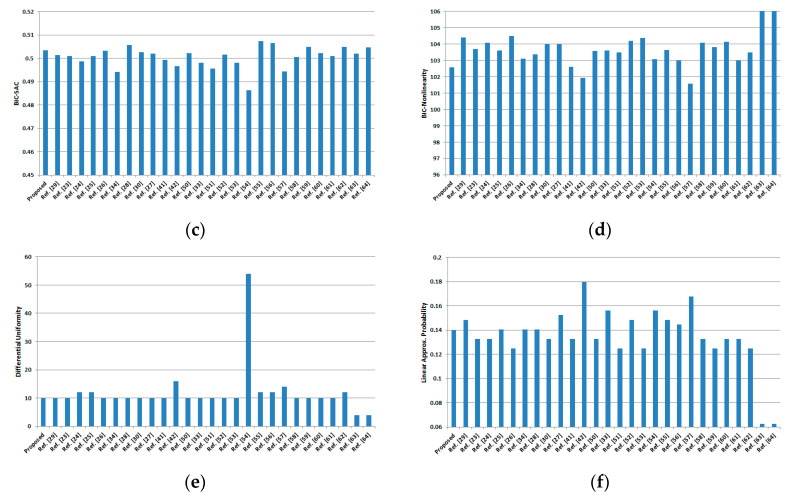
Graphical comparison analyses of 8 × 8 S-boxes under (**a**) nonlinearity, (**b**) SAC, (**c**) BIC–SAC, (**d**) BIC-nonlinearity, (**e**) DU and (**f**) linear approximation probability (LAP).

The nonlinearity performance of the proposed S-box comes out to be outstanding, as all three statistics, namely the minimum (110), maximum (112), and average (111.25), are significantly higher than the S-boxes in Table 7. The same comparison is also shown graphically in Figure 2a. Thus, highly nonlinear S-boxes can be constructed by using the proposed novel scheme.

The proposed S-box was also found to show good SAC behavior, as our SAC value of 0.5007 is quite better than the scores of S-boxes constructed in References [23,24,25,26,27,28,30,33,34,41,42,50,51,52,53,54,55,56,57,58,59,60], as evident from Table 7 and Figure 2b. The proposed S-box satisfies the SAC criterion quite diligently as compared to other contemporary S-boxes.

The results of bits independence criterion show that the proposed S-box also exhibits acceptable BIC performance, as the BIC–SAC and BIC-nonlinearity scores are satisfactory, as shown in Figure 2c,d.

The differential uniformity of our S-box is only 10, which is less and better than S-boxes investigated in References [24,25,42,54,55,56,57], and it is comparable to other S-boxes in terms of robustness to differential cryptanalysis. The DU comparison is also shown graphically in Figure 2e.

A block cipher can withstand the linear cryptanalysis if the employed S-box is dynamic, highly nonlinear, and linear approximation probability is low. The LAP score of 0.14025 is obtained for the proposed S-box, which shows a satisfactory value. Moreover, this probability score is quite lower and better than the LAP score of the S-boxes available in References [25,27,28,29,33,34,42,52,54,55,56,57]. The LAP comparison is also shown graphically in Figure 2f.

### 4.7. Time Analysis

The computation time is one of the essential features for any security application. In order to have an idea of time consumption of our proposed scheme, we calculated the time taken by the scheme to evolve an S-box. It was found that the scheme takes a very nominal amount of time, (on average) only 3.5459 s. This computational time is considerably nominal compared to many optimization-based evolution of S-boxes available in the literature, such as References [5,8,38,53].

### 4.8. S-Box Validation for Image Encryption Applications

**Figure 3:**
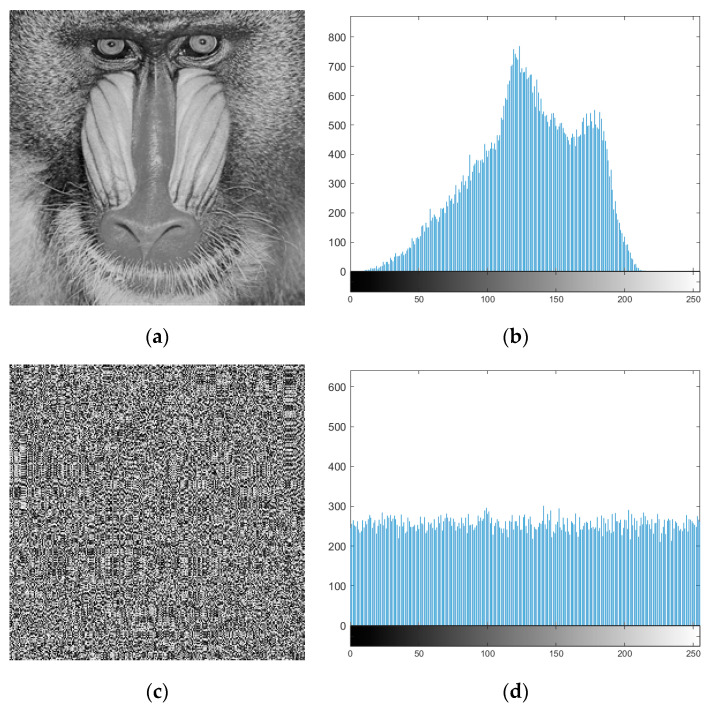
Results of image encryption using proposed S-box for *Baboon* image (**a**) plain-image, (**b**) histogram of plain-image, (**c**) encrypted image, and (**d**) histogram of encrypted image.

In recent days, the strong S-boxes have been predominantly utilized for image encryption applications. It is prudent to validate the appropriateness of proposed S-box for such security application. Accordingly, we applied the S-box presented in this study, to encrypt the standard 8-bit encoded Baboon plain-image. The encryption involves the forward and reverse substitution using the proposed S-box. The results of encryption along with the distribution of pixels in the respective images are shown in Figure 3. The obtained encrypted image shows high visual distortion and good encryption effect. To quantify the encryption performance exhibited by our S-box, we evaluated the statistical tests, such contrast, correlation, energy, and homogeneity, which are members of the Majority Logic Criteria (MLC) suite. The description and details of these tests are available in author’s previous studies [7,40,48]. The statistical scores of encryption performance under MLC analysis are listed in Table 8 and compared with encryption performances of S-boxes investigated in References [48,61]. The obtained results for MLC analysis validate the appropriateness of proposed S-box for image encryption applications.

## 5. Conclusions

Highly nonlinear substitution-boxes provide good nonlinear transformation and confusion of input plaintext data to generate ciphertext data in block cryptosystems. Such S-boxes are also potent to offer great resistance to mitigate the linear and other types of attacks which may exploit the existence of linearity in the security system. Moreover, the key-dependent and dynamic S-boxes also tend to provide more strength to the cryptosystem. This paper has proposed a novel scheme of constructing dynamic and highly nonlinear S-boxes. Our scheme is based on the dynamics of two-state time-delayed fractional-order Hopfield neural network system. Firstly, the anticipated scheme generates an initial S-box which is made to evolve for high nonlinearity, using the heuristic. The proposed scheme and constructed S-box possessed high cryptographic strength and large key space. It was found that the proposed scheme is able to evolve an S-box with nonlinearity of 111.25, strict avalanche criteria value of 0.5007, and differential uniformity of 10. The comparison analysis with some available dynamical-systems-based S-box methods and others validated the better performance of our S-box.

As future directions of the presented study, we can also check the effectiveness of proposed scheme in generating large-size *n*x*n* S-boxes (*n* > 8). The large-size S-boxes have better potential to offer resistance to several attacks than small-size S-boxes. The generation of large-size S-boxes is rarely investigated in the literature. Moreover, the evolution is initial S-box is based on only lone criteria of nonlinearity. The evolution process can be executed to satisfy multiple S-box performance parameters.

## Figures and Tables

**Table 1:** Experimental settings for simulation.

System (1)				System (2)		
parameter		value		parameter		value
*x*_1_(0)		2.1		*y*_1_(0)		2.3
*x*_2_(0)		3.7		*y*_2_(0)		8.9
*p*_1_		0.12		*p*_2_		0.12
*q*_1_		0.3		*q*_2_		0.67
*τ*_1_		0.1		*τ*_2_		0.33
*h*		0.005		Δ*τ*		0.05
*N*		200		*itr_max*		2000
*step_x*		57		*step_y*		31
*prime*_1_		23		*prime*_2_		37

**Table 2:** Proposed 8 × 8 substitution-box.

127	180	219	232	97	108	195	39	104	136	169	49	189	143	81	240
191	163	134	21	200	66	23	202	89	225	241	50	61	15	93	201
98	161	14	205	42	12	102	68	230	130	144	149	10	78	183	80
4	26	70	137	41	199	171	142	154	55	101	111	18	208	31	7
168	133	30	103	190	253	250	92	218	212	198	28	54	9	27	118
105	83	76	187	69	238	235	246	52	158	155	29	64	156	11	20
135	252	173	179	176	220	99	244	231	138	128	165	254	6	203	77
194	177	87	82	84	37	170	204	239	227	150	247	17	85	117	107
57	215	210	79	75	186	91	131	25	162	152	147	175	151	139	193
224	251	96	40	164	207	213	62	119	109	122	214	114	132	248	223
206	19	159	67	88	63	222	60	110	71	112	16	167	116	46	45
38	221	72	100	129	209	228	157	146	115	243	3	140	8	145	86
0	106	192	166	90	65	153	125	5	184	160	245	58	113	44	196
178	121	141	185	226	197	56	43	181	32	94	13	182	120	51	249
229	188	47	216	255	59	234	124	48	217	53	126	35	73	22	95
24	237	242	36	236	1	211	74	148	172	174	123	34	33	233	2

**Table 3:** Dependency matrix for strict avalanche criterion (SAC).

0.5312	0.4531	0.5	0.5625	0.5625	0.5	0.5312	0.4843
0.5937	0.4375	0.5156	0.5312	0.4531	0.5	0.4843	0.5468
0.4687	0.4687	0.5468	0.5156	0.5312	0.4843	0.5	0.4687
0.4531	0.4843	0.5	0.5312	0.4531	0.5	0.4843	0.5312
0.5312	0.5312	0.4843	0.5312	0.4375	0.5	0.5468	0.4843
0.5468	0.4687	0.5	0.5312	0.4375	0.4687	0.5156	0.4531
0.5156	0.4062	0.5781	0.5468	0.4843	0.5	0.5	0.5
0.4843	0.4843	0.5	0.4062	0.5312	0.4843	0.5781	0.4687

**Table 4:** Bits independence criterion (BIC) results for nonlinearity.

0	104	100	104	100	98	102	98
104	0	100	104	106	108	104	104
100	100	0	104	104	108	104	98
104	104	104	0	104	100	104	104
100	106	104	104	0	106	102	106
98	108	108	100	106	0	96	102
102	104	104	104	102	96	0	98
98	104	98	104	106	102	98	0

**Table 5:** BIC results for SAC.

0	0.5175	0.5136	0.5078	0.5039	0.5039	0.5	0.5136
0.5175	0	0.5234	0.5214	0.4843	0.5156	0.4882	0.5097
0.5136	0.5234	0	0.5097	0.5039	0.4902	0.5	0.4726
0.5078	0.5214	0.5097	0	0.4882	0.4921	0.4863	0.5039
0.5039	0.4843	0.5039	0.4882	0	0.5195	0.5117	0.5214
0.5039	0.5156	0.4902	0.4921	0.5195	0	0.5078	0.5117
0.5	0.4882	0.5	0.4863	0.5117	0.5078	0	0.4726
0.5136	0.5097	0.4726	0.5039	0.5214	0.5117	0.4726	0

**Table 6:** Differential matrix for differential uniformity (DU).

10	6	8	4	8	8	6	10	6	6	8	6	6	8	6	6
6	8	6	6	8	6	6	6	8	6	8	8	6	6	8	8
8	6	6	8	8	6	4	6	6	6	8	4	8	6	6	6
6	6	8	8	6	6	6	6	6	8	6	8	6	10	6	6
6	6	6	6	8	6	10	6	6	6	6	6	8	8	6	6
6	6	6	4	6	6	6	6	6	6	6	6	6	6	6	10
8	6	6	6	6	6	6	8	6	8	6	6	6	8	4	8
6	6	8	8	6	6	6	6	8	6	8	6	6	6	6	10
6	6	8	8	6	8	6	6	6	6	6	6	6	6	6	8
8	6	6	6	8	8	8	6	8	6	8	8	8	8	6	8
6	6	8	6	6	8	6	8	8	6	8	8	8	8	6	6
6	6	8	6	6	6	6	4	6	8	6	6	6	6	8	6
6	6	6	6	4	8	6	6	8	8	6	6	8	8	6	4
8	4	6	6	6	6	8	6	6	8	6	6	6	6	8	6
6	4	6	6	8	6	6	6	6	6	8	8	6	6	6	6
6	6	6	8	8	6	6	6	10	6	6	6	6	8	6	0

**Table 7:** Performance comparison of 8 × 8 S-boxes.

S-box	Nonlinearity			SAC	BIC–SAC	BIC-NL	DU	LAP
	*min*	*max*	*mean*					
Proposed	110	112	111.25	0.5007	0.5034	102.57	10	0.1403
Reference [23]	100	106	103.2	0.5048	0.5009	103.7	10	0.1328
Reference [24]	98	108	103	0.5012	0.4988	104.07	12	0.1328
Reference [25]	100	108	104.5	0.4978	0.5009	103.6	12	0.1406
Reference [26]	104	108	105.80	0.4976	0.5032	104.5	10	0.1250
Reference [27]	106	110	108	0.5073	0.502	104	10	0.1523
Reference [28]	104	110	106	0.5039	0.5058	103.38	10	0.1406
Reference [29]	102	108	106	0.5002	0.5013	104.4	10	0.1484
Reference [30]	106	110	108.5	0.5017	0.5026	104	10	0.1328
Reference [33]	103	109	105.1	0.5061	0.4982	103.6	10	0.1563
Reference [34]	100	108	104.7	0.4982	0.4942	103.1	10	0.1406
Reference [41]	102	108	105.25	0.5037	0.4994	102.6	10	0.1328
Reference [42]	84	106	100	0.4812	0.4967	101.93	16	0.1796
Reference [50]	104	108	106.75	0.4076	0.5022	103.57	10	0.1328
Reference [51]	106	108	106.7	0.4941	0.4957	103.5	10	0.125
Reference [52]	106	110	107	0.5014	0.5016	104.2	10	0.1484
Reference [53]	106	108	107.5	0.4943	0.4982	104.36	10	0.125
Reference [54]	96	106	103.25	0.5151	0.4864	103.07	54	0.1562
Reference [55]	104	108	106.75	0.5031	0.5074	103.64	12	0.1484
Reference [56]	105	107	106	0.5066	0.5065	103	12	0.1445
Reference [57]	98	108	102.3	0.4836	0.4944	101.57	14	0.1679
Reference [58]	106	108	106.5	0.5009	0.5005	104.07	10	0.1328
Reference [59]	104	110	106.25	0.4990	0.5050	103.8	10	0.125
Reference [60]	106	108	106.5	0.5046	0.5023	104.14	10	0.1328
Reference [61]	94	108	103.5	0.5024	0.5009	103	10	0.1328
Reference [62]	96	108	102.25	0.5059	0.5050	103.5	12	0.125
Reference [63]	112	112	112	0.5058	0.502	112	4	0.0625
Reference [64]	112	112	112	0.5058	0.5046	112	4	0.0625

* Where, NL stands for nonlinearity.

**Table 8:** Majority Logic Criteria (MLC) analysis results for image encryption using proposed S-box.

Baboon Image	Contrast	Correlation	Energy	Homogeneity
Plain-image	0.63265	0.7983	0.09438	0.78209
Encrypted image	10.4227	0.0064	0.01566	0.39483
Reference [48]	10.4291	−0.0128	0.0157	0.3889
Reference [61]	10.3986	0.0072	0.0158	0.4214
